# Evaluation of Different Benzimidazole Formulations Against Sheep Naturally Infected with *Fasciola hepatica* and Anthelmintic Resistance Analysis

**DOI:** 10.3390/vetsci13020205

**Published:** 2026-02-20

**Authors:** Laura González del Palacio, Matthew James Denwood, Elora Valderas-García, Verónica Castilla-Gómez de Agüero, Rafael Balaña-Fouce, María Martínez-Valladares

**Affiliations:** 1 Instituto de Ganadería de Montaña (CSIC-Universidad de León), Grulleros, 24346 León, Spain; lgonzp@unileon.es (L.G.d.P.); elora.valderas@csic.es (E.V.-G.); 2 Departamento de Sanidad Animal, Facultad de Veterinaria, Universidad de León, Campus de Vegazana, 24071 León, Spain; 3 Department of Veterinary and Animal Sciences, University of Copenhagen, 1870 Frederiksberg, Denmark; md@sund.ku.dk; 4 Instituto de Parasitología y Biomedicina López Neyra, Armilla, 18016 Granada, Spain; veronica.castilla@ipb.csic.es; 5 Departamento de Ciencias Biomédicas, Facultad de Veterinaria, Universidad de León, Campus de Vegazana, 24071 León, Spain; rbalf@unileon.es

**Keywords:** sheep, *Fasciola hepatica*, anthelmintic resistance, benzimidazoles, faecal egg count reduction test

## Abstract

Fasciola infection, which is widely distributed and has a major impact on livestock production, is emerging as a significant zoonotic parasitic disease affecting both human and animal health worldwide. The main control strategy currently relies on a limited number of anthelmintic drugs, especially benzimidazoles such as albendazole and triclabendazole. The aim of this study was to evaluate the efficacy of albendazole and triclabendazole in a sheep flock in northwestern Spain naturally infected with *F. hepatica* and to test fenbendazole and oxfendazole as alternative formulations. For this purpose, a faecal egg count reduction test (FECRT) was conducted applying various statistical methods based on the guidelines used for gastrointestinal nematodes. This study represents the first application of the new classification framework for *F. hepatica*, emphasizing the urgent need for standardized resistance detection protocols for this parasite and underlining the growing challenge of anthelmintic resistance in the treatment of fasciolosis.

## Simple Summary

Fasciola infection is now a widely known parasitic disease that affects animals and humans, causing significant losses in livestock production worldwide. Its control depends mainly on a few antiparasitic drugs, such as albendazole and triclabendazole. The aim of this study was to evaluate the efficacy of these drugs in a flock of sheep in northwestern Spain infected with the species *Fasciola hepatica*, while also testing other drugs from the same family as an alternative to these treatments and comparing the results using different analysis methods. Thanks to this comparison, which was carried out using a technique that counts the eggs eliminated in the animals’ faeces at different times, it was possible to demonstrate that resistance to antiparasitic drugs is a growing problem in the treatment of fasciolosis, as well as the urgent need to standardize the methods currently used to detect such resistance.

## 1. Introduction

Fasciolosis is a significant parasitic disease caused by parasites of the genus *Fasciola*. This includes the species *Fasciola hepatica*, which has a very wide geographic distribution, possibly the widest of any helminth parasite, being present in all continents except Antarctica [1]. The socioeconomic and medical importance of Fasciola infection is unquestionable. The global agricultural community is widely affected by this trematode, which can infect ruminants worldwide and cause huge economic losses. The disease is considered a serious animal health problem in many rural and urban areas of the world [2], and it is also now emerging as a major zoonosis. It is classified by the World Health Organization as a neglected tropical disease with an estimated 17 million people infected [3] and a further 180 million people at risk of infection [4]. Its significant impact on agriculture and human health together with the increasing demand for animal-derived food products to support global population growth has led fasciolosis to become a major One Health problem [5]. *F. hepatica* has special implications in ruminants such as sheep and cattle as it can limit animal production by reducing the growth rate, feed conversion, milk and meat production, and even the quality and quantity of wool in sheep [6]. The condemnation of livers in slaughterhouses, cost of treatments, reduced fertility, possible secondary infections, and deaths in acute cases appear to be direct damage derived from the infection [7]. Sheep are particularly vulnerable to acute infections due to a lack of effective immune regulation, according to previous studies [8,9]. Clinical signs of acute infection include weight loss, mucosal pallor, apathy, depression, muscle tremors, drooling, and even death. Acute fasciolosis develops six to eight weeks after the ingestion of large numbers of infective metacercariae. The migration of flukes leads to liver damage and blood loss, causing anaemia and proteinemia. In sheep, this can frequently lead to death. Although sudden death can occasionally be seen in cattle, sheep are more susceptible to acute fasciolosis [8].

The diagnosis of *F. hepatica* infection in European livestock has increased over the last 15–20 years, possibly due to climate changes, variations in farming practices (including animal movement and land use), and the emergence of resistance [2]. In this respect, Martínez-Valladares et al. [10] reported a significant increase in Fasciola infection prevalence in sheep flocks in the area under study, rising from 26.7% in the early 1990s to 60.5% in 2011, which is likely associated with the expansion of irrigated areas. Furthermore, the genetic diversity of different *Fasciola* spp. strains is a factor to be considered in the parasite’s further spread [11].

Control strategies for Fasciola infection rely heavily on the use of anthelmintic drugs, of which there are only a limited number [12]. Considering the restricted range of drugs available, the development of anthelmintic resistance (AR) in *Fasciola* spp. is concerning [6]. One of the most important chemical groups among all flukicides is benzimidazoles (BZs). These are broad-spectrum anthelmintic compounds that are widely used in human and veterinary medicine to control helminth infections. This large family includes not only albendazole (ABZ) and mebendazole but also triclabendazole (TCBZ), flubendazole, fenbendazole (FENB), oxfendazole (OXF), thiabendazole, and oxibendazole [13]. ABZ is one of the recommended BZs for the control of Fasciola infection. This drug appears to have an ovicidal effect on *F. hepatica* [2], and it is well tolerated, affordable, and effective against >12-week-old liver flukes, with an activity higher than 90% [14,15]. However, Fasciola infection is primarily treated with TCBZ due to its ability to act against both immature and mature stages [16,17], with even an activity of 100% against mature flukes [18,19]. TCBZ has been used since the 1980s, demonstrating good activity against *Fasciola* spp. over many years [16,20,21]. Unfortunately, resistance of *F. hepatica* to TCBZ was first reported in Australian sheep in 1995, and in recent years this liver fluke has become resistant to the drug in several countries; reports of TCBZ resistance include unrelated geographical regions including Northern Ireland, Scotland, Wales, the Republic of Ireland, the Netherlands, Spain, mainland Europe, Russia, Australia, New Zealand, Peru, and Argentina, among others [22]. The high frequency of TCBZ use as monotherapy is a major contributor to the development of resistance [23,24]. The levels reached have not been as pressing as those seen with other anthelmintics (including other benzimidazoles), but it remains a cause for concern due to the dependence of the livestock industry on antiparasitic drugs to preserve animal health. However, TCBZ marketing varies greatly between countries, which poses a challenge in terms of its use as the treatment of choice against *F. hepatica*. In Spain, in fact, it is not currently marketed.

Several methods have been described to evaluate AR in ruminants. One well-known in vivo method used to assess anthelmintic efficacy in the field is the faecal egg count reduction test (FECRT). This test is an important tool for monitoring the development of AR [25]. Based on the reduction in the number of eggs in faeces after anthelmintic treatment [26], it allows the evaluation of drug efficacy for all types of anthelmintics, in all animal species, and for multiple parasite species, without the need to sacrifice the animals. It can also be performed locally, without the need for a reference diagnostic laboratory or specialized equipment and/or expertise [27]. However, to confirm the resistance, post-mortem examination is also recommended [28] in treated animals, the controlled efficacy test (CET), or “dose and slaughter trial”, which was not allowed in the present study. Fasciola infection can also be confirmed by detecting antigens in faeces, known as coproantigens, which enables diagnosis even during the pre-patent period. Moreover, monitoring the reduction in coproantigen levels after anthelmintic treatment provides a useful tool for assessing treatment efficacy and identifying potential anthelmintic resistance [29,30].

Several guidelines have been published for the detection of AR in gastrointestinal nematodes (GINs) [26,27,31]. However, to date, there are no guidelines for the detection of resistance in *Fasciola* spp. In this context, we tested the efficacy of ABZ and TCBZ, along with other BZs as alternative formulations, in a flock that was naturally infected with *F. hepatica*. To quantify the efficacy of the drugs, we applied different statistical methods in combination with the classification framework recommended in the latest of the aforementioned guidelines for GINs [27]. This is the first time that this statistical framework has been used to classify the efficacy of anthelmintics against *F. hepatica*.

## 2. Methods

### 2.1. Description of the Sheep Flock

This study was conducted on a flock of sheep with a history of fasciolosis and suspected ABZ resistance. The farm was located in Villaumbrales, a town in the autonomous community of Castilla y León, in northwest Spain. The sheep were of the Castellana breed and were reared for milk and meat production. The flock grazed on irrigated pastures for 6–8 h daily and was housed indoors at night. Grazing was only occasionally suspended on days of severe weather (heavy rainfall or snowfall). The last anthelmintic treatment with ABZ had been administered at least six months before the start of this study.

This study was divided into three trials (T1, T2, and T3). According to the treatment schedule, ABZ and OXF efficacy were measured in T1 at different doses, followed by the measurement of FENB efficacy in T2 and TCBZ efficacy in T3.

### 2.2. Preselection of Naturally Infected Animals

Before starting the trials, individual analysis was carried out in 82 sheep to determine the number of *F. hepatica* eggs per gram (epg) in faeces in sheep randomly selected from the same flock. Faecal samples were taken directly from the rectum using plastic gloves and analysed by a simple sedimentation method [32]. Each of the hemichambers of a McMaster slide was filled to count the eggs, under a light microscope using 40× magnification. Faecal eggs were counted in a total volume of 1 mL (0.5 mL per hemichamber), and the count values were obtained by multiplying the total number of eggs by 15 (multiplication factor).

### 2.3. Faecal Egg Count Reduction Test

Each sheep was visually inspected before treatment to determine general health status and weighed in all trials, in order to calculate the individual drug dose. All sheep involved in these trials were maintained under the same conditions as the rest of the flock throughout the study.

Of particular concern is the absence of standardized guidelines or validated diagnostic tests for drug resistance in Fasciola infection. In this study, a two-week interval between pre- and post-treatment sample collection was established following the literature for the FECRT [33,34,35,36,37].

In T1, four groups each of 10 positive animals were established. Each group was orally treated with an anthelmintic at the following doses: ABZ at 7.5 mg/kg body weight (bw) (Ganadexil^®,^ oral liquid), OXF at 5 mg/kg bw (Bovex^®^ 2.265% *w*/*v*, Chanelle Pharma, Galway, Ireland), OXF at 30 mg/kg bw (Bovex^®^ 2.265% *w*/*v*, Chanelle Pharma, Galway, Ireland, oral liquid), and a control group treated with water; all treatments were administered by oral route. Subsequently, in T2, the efficacy of FENB at a dose of 10 mg/kg bw by oral route (Panacur^®^10%, Intervet Productions S.A., Igoville, France, oral liquid) was evaluated in a group of 19 sheep. This group was compared to a control group of 10 animals that were administered 10 mL of tap water. In T3, 28 animals were treated with TCBZ by oral administration at a dose of 10 mg/kg bw (Biofasiolex^®^ T10, Biogénesis-Bagó, Buenos Aires, Argentina, oral liquid). In this trial, no control group was included.

In the three trials, individual faecal samples were taken from all sheep on the day of treatment and 14 days post-treatment (pt) to measure the *F. hepatica* epg by a sedimentation method and then calculate the FECRT.

Four methods were selected for the calculation and interpretation of the faecal egg reduction (FECR) in order to detect AR. Three of these methods are based on the guideline recently published by Kaplan et al. [27] for the detection of AR in GINs infecting ruminants. This guideline recommends using two web-based analysis tools to detect AR, both of which were used in the current study: (i) the eggCounts web interface (URL: http://shiny.math.uzh.ch/user/furrer/shinyas/shiny-eggCounts/, accessed on 6 March 2024), which is based on a method using a Bayesian hierarchical model [38,39]; (ii) the FECRT web interface (URL: http://www.fecrt.com, accessed on 6 March 2024), which implements several different analysis methods including the Delta method [40] and the WAAVP method [26,41], which can be pre-configured with parameter values following the specifications previously described by Denwood et al. [25] and Kaplan et al. [27]. Therefore, in this study, the “eggCounts” web interface model was used as the first method, the “Delta method” as the second model, and the “WAAVP method” as the third calculation model. The fourth method is a customised Bayesian model similar to that presented by Denwood et al. [42] for paired data and Geurden et al. [43] for unpaired data and implemented in JAGS [44], a program for Bayesian graphical modelling which aims for compatibility with Classic BUGS (acronym for *Bayesian inference Using Gibbs Sampling*) using the runjags package [45] for R [46]. Regardless of the method, the FECR can be performed on the basis of measuring pre- and post-treatment FEC of the same animals using a “paired study design” or by measuring post-treatment FEC in both the treated and control groups, using an “unpaired study design”. In addition, according to Kaplan et al. [27], there are two different ways to design the experiments: (i) a version intended for use in scientific studies (the “research protocol”), designed to detect small variations in drug efficacy, and (ii) a version with less stringent experimental requirements (the “clinical protocol”) intended for use by veterinarians and farmers to detect larger modifications in efficacy. The basis for the difference between the “clinical protocol” and the “research protocol” is the width of the “grey zone”, which is determined by the lower threshold of efficacy and derives from the range of observed efficacies within which we can expect an inconclusive result from the FECRT. This grey zone helps to detect small reductions in efficacy. The expected efficacy is considered to be 99% for all anthelmintics against GINs of sheep [27]. It is also worth noting that the term “lack of efficacy” was used rather than “resistance” in the case of OXF and FENB because these drugs are not currently registered and/or recommended against *F. hepatica*.

### 2.4. Statistical Analysis

All data handling and statistical analyses were performed in R version 4.4.1 [46]. For T1 and T2, the data were analysed as unpaired and/or paired studies using the four different approaches described previously. For all of them, an expected efficacy of 99% and a lower efficacy threshold of 95% (choosing the research protocol with a grey zone of 95–99% and accepting a 5% type 1 error rate) were applied to calculate the FECR upper and lower confidence intervals (CIs) [27]. In T3, without a control group, a paired study design was applied, following the same methods. Since the recent guidelines [27] only use the range of the 90% CIs to classify efficacy, all data were expressed as a range of CIs and the classification of efficacy based on this rather than observed FECR estimate.

Regarding ABZ and TCBZ, and following the WAAVP guidelines [27], resistance was confirmed when the upper 90% CI was less than the expected efficacy (corresponding to the upper limit of the grey zone). Susceptibility was determined when the lower 90% CI was greater than or equal to the lower efficacy threshold (corresponding to the lower limit of the grey zone), and the upper 90% CI was greater than or equal to the expected efficacy (corresponding to the upper limit of the grey zone). The result was deemed to be inconclusive when neither of the criteria were met. For FENB and OXF, the same approach was followed, but in this case, the results were interpreted as “effective” or “reduced efficacy” or “inconclusive”.

## 3. Results

**Figure 1:**
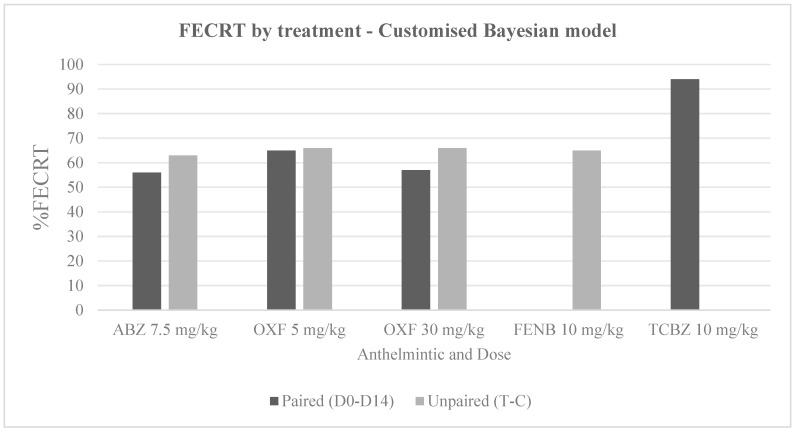
Comparison of FECRT (%) by treatment using a customised Bayesian model. For each anthelmintic treatment, FECRT values obtained under paired (D0–D14) and unpaired (T–C) experimental designs are shown. Absence of bars reflects unavailable data rather than zero values.

**Figure 2:**
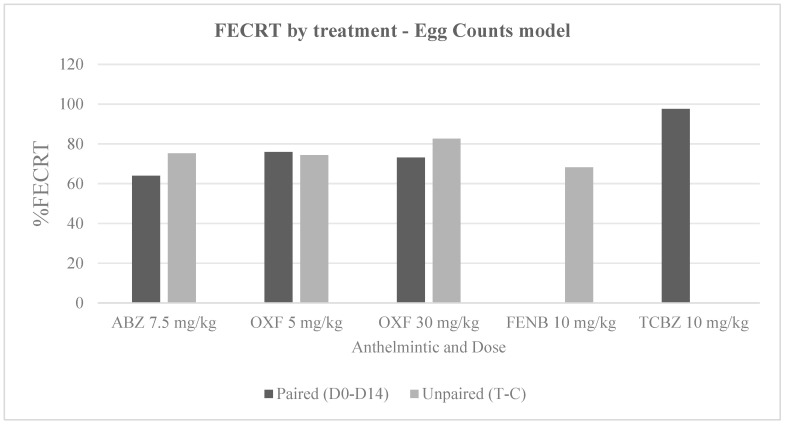
Comparison of FECRT (%) by treatment using the Egg Counts model. Bars represent paired (D0–D14) and unpaired (T–C) designs within each treatment. Absence of bars reflects unavailable data rather than zero values.

Before conducting T1, 82 randomly selected sheep had a mean of 82 epg (0–915 epg), and from these animals, 24 sheep with the highest FEC were selected for this trial, with a mean of 258 epg (150–915 epg). Regarding the FECR for ABZ, the mean FEC on day 0 was 78 (30–165 epg), and this value dropped to 29 epg (0–105 epg) on day 14 pt. FECRT results ranged between 56.0 and 75.3%. Table 1 illustrates the mean values mentioned above and detailed FECRT percentages, whereas Table 2 shows the FECR CIs which revealed “resistance” to ABZ for both paired and unpaired study designs according to the different approaches. Figure 1 and Figure 2 show a comparison of the FECRT (%) estimates for each treatment according to the customised Bayesian and Egg Counts models, respectively.

Screening OXF at 5 mg/kg revealed a mean of 113 epg on day 0 pt (45–240 epg) and 28 epg (0–90 epg) on day 14 pt (see Table 1), with FECRT percentages ranging from 65 to 75.9% (see Table 2). Regardless of the method used to measure the efficacy, FECR CIs revealed “reduced efficacy”. When the drug was tested at a dose five times higher than the previous one (30 mg/kg), the mean FEC decreased from 74 epg (15–165 epg) to 21 epg (0–120 epg) at 14 days pt (see Table 1), and the FECRT measured values from 57.0 to 82.6%. According to the classification, OXF resulted in “reduced efficacy” for both paired and unpaired study designs. According to the Delta method, WAAVP method, customised Bayesian model method, and standard Bayesian model methods, OXF also showed “reduced efficacy” after testing both doses (see Table 2).

For FENB at 10 mg/kg (T2), the mean FEC on day 14 pt was 265 epg (30–825 epg) in the treated group and 819 epg (435–1300 epg) in the control group (see Table 1). The FECRT range waved between 65.0 and 68.2% (see Table 2). Considering the design of this trial as unpaired, FENB resulted in “reduced efficacy”, regardless of the method used.

Prior to conducting T3, 57 sheep were randomly selected, with an average of 26 epg (0–165 epg); of these, 28 sheep with the highest values were included in the trial to test the TCBZ at a dose of 10 mg/kg. The mean FEC at day 0 pt was 44 epg (15–255 epg), and this value decreased to 1 epg (0–30 epg) at day 14 pt (see Table 1). The measured percentages for the FECRT were 94.0% and 97.6% (see Table 2). For this unpaired trial, the result was “inconclusive” for all four approaches, as the lower 90% CIs were below the lower efficacy threshold value of 95% in each case. However, this result relies heavily on a single animal with a non-zero FEC post-treatment; since all of the animals in this trial had zero eggs after treatment, except for one individual with two eggs counted after treatment, we cannot exclude the possibility that these two eggs were stored in the gall bladder, meaning the animal was actually free of infection. This would suggest that the efficacy should be 100%, which in this case would result in a conclusion of susceptible rather than inconclusive. Future recommendations should specifically address this particular *F. hepatica* challenge.

## 4. Discussion

Fasciolosis remains one of the most significant helminthic diseases affecting livestock in many countries in the world. There is an urgent need to find alternatives that can address the changing climate, the increasing prevalence of infection, and resistance to the limited number of available flukicides.

Currently, due to the fact that the majority of the control of Fasciola infection in animals relies on the use of anthelmintic drugs [47], and especially those in the BZ family, evaluation of the efficacy of these drugs in ruminants infected by helminths is a key factor in avoiding the development and spread of resistance. As TCBZ is not marketed in Spain, ABZ is one of the most widely used drugs and is also broadly used for the control of GINs. The sheep flock in the present study had a history of recurrent fasciolosis and was therefore suspected to be resistant to ABZ. According to the guidelines described by Kaplan et al. [27], resistance to ABZ was confirmed in the adult stage of *F. hepatica* in the present study, as the upper limit of the grey zone was found to be less than the expected efficacy for all four approaches (see Table 1). Moreover, FECRT values ranged from 56.0 to 75.3%. The genetic diversity of fluke populations can indicate that selection for drug resistance is likely to occur frequently, as well as the fluke’s response to other environmental pressures, such as climate change [48]. In accordance with our results, it is assumed that there are differences in the mechanisms of action of the two drugs (ABZ and TCBZ), which would explain the absence of side resistance between them in the current study, despite them belonging to the same BZ family. Side resistance is defined as the phenomenon whereby parasites resistant to a drug of one chemical class are also resistant to others of the same class [49]. To illustrate this, other studies have reported cases of ABZ-resistant but TCBZ-susceptible *F. hepatica* in countries such as Argentina and Sweden [50,51,52], as well as active ABZ susceptibility against a TCBZ-resistant isolate of *F. hepatica* [53].

With the focus of providing other therapeutic options for treating *F. hepatica*, OXF and FENB were also evaluated to determine their possible range of efficacy. In T1, OXF was tested at two different doses (5 and 30 mg/kg bw) against a control group. At a dose of 5 mg/kg bw, OXF is widely used against GINs in sheep, achieving high therapeutic efficacies (97.69% —CI95% between 93.38 and 99.20%— and 99.13% —CI95% between 96.59 and 99.78%—) [54]. Furmaga et al. [55] evaluated OXF against sheep infected with *F. hepatica* at doses of 5 and 15 mg/kg bw, reporting reductions in the fluke burdens up to 78% (CI 59–100%) and 70%, respectively. These reductions are in closer agreement with the results of the current study. In T1, the FECR revealed a lack of efficacy against *F. hepatica* infection in the OXF-treated group at 5 mg/kg bw (FECRT: 65–75.9%, CIs from 29.4 to 92.8%). Also, Khan et al. [56] tested OXF at 22.65 mg/kg with an efficacy of 72.85% against *F. hepatica*. In a masked, controlled study, Gómez-Puerta et al. [57] demonstrated a significant difference in the FEC (*p* < 0.01) between control and treated groups of naturally infected sheep after a treatment of 30 mg/kg of OXF by oral route against *F. hepatica*; no side effects were discernible in the OXF-treated animals. In the present study, despite the alternative treatment with OXF (at 30 mg/kg) improving control of the infection (FECRT: 57.0–82.6%), the upper 90% CIs were all less than the expected efficacy.

FENB also has anthelminthic activity [58], as it is a metabolite of OXF. The efficacy of this drug at dose levels of 5 mg/kg bw and 7.5 mg/kg bw was previously tested against natural infections of *Fasciola* species by Güralp and Tınar [59]. That study revealed 23% and 92% of efficacy for *F. gigantica*, respectively, and 28% for *F. hepatica* at 7.5 mg/kg. In the present study, the efficacy of the FENB at 10 mg/kg achieved CIs between 47 and 81%, therefore resulting in the upper 90% CI being less than the expected efficacy and the FECRT values being from 65.0 to 68.2%.

The CET, or “dose and slaughter trial”, is the most reliable diagnostic method for the detection of resistance. However, it is unlikely that this can be carried out under field conditions as it involves the sacrifice of the animals included in the study. According to the WAAVP guidelines for evaluating the efficacy of anthelmintics in ruminants [60], necropsies conduced within the CET should be performed 2–3 weeks after treatment. Therefore, despite its limitations, the most widely used test for detecting resistance in vivo is the FECRT, despite its limitations. FEC data alone may not be entirely accurate, particularly when fluke burdens are low. For this reason, it is advisable to perform the coproantigen reduction test simultaneously, although this was not possible in the present study. According to Fairweather et al. [1], the FECRT is typically performed comparing the FEC on the day of treatment and then later at 3 weeks post-treatment, although there are no guidelines or validated tests for diagnosing drug resistance. In fact, as described in Section 2, numerous studies consider two weeks to be the reference period for calculating the FECRT. In this study, the reduction was calculated at 2 weeks post-treatment since one of the main objectives was to determine the resistance of ABZ and other formulations against mature flukes. As efficacy can be determined in the CET from the second week post-treatment, this was also the case in vivo. Had the FECRT been performed in the third week pt, there would have been a risk of detecting eggs in the faeces from immature flukes and not only from mature ones, especially considering that the animals in the current study were never kept in stables and therefore went out to pasture for a few hours every day and were kept indoors at night. Therefore, at the time of treatment, the animals could be infected with both mature and immature flukes. Specifically in the case of TCBZ, which is effective against both mature and immature forms, resistance could be assessed later (at 3 weeks pt). This interval enables the evaluation of its effect on immature final stages and ensures the elimination of residual eggs remaining in the gallbladder due to the irregular *F. hepatica* egg shedding. In the present study, however, the priority was to evaluate the efficacy of ABZ and other BZ formulations (as TCBZ was not marketed in Spain) against mature forms; therefore, a uniform assessment time of 2 weeks pt was applied to all groups. On the other hand, and as mentioned above, resistance can also be confirmed using methods such as coproantigen reduction; however, a limitation of the present study is that this test could not be performed. Nonetheless, the FECRT results clearly demonstrated resistance to ABZ, making the use of an additional confirmatory method less critical in this case. With respect to the different statistical methods used for the evaluation of anthelmintic efficacy in terms of FECR, it is important to highlight that no standard protocols or specific guidelines have been established for *Fasciola* spp. Previous WAAVP methods [26] used to calculate the FECR in GINs recommend an unpaired design using a non-treated control group; however, according to the new WAAVP guidelines [27], the use of a paired study design increases statistical power for detecting resistance compared to the use of a control group in an unpaired study design [61]. Furthermore, parallel studies conducted for the new guidelines have shown that paired study designs yield more conclusive outcomes than unpaired study designs. Using the new criteria given for the interpretation of FECR results, and eliminating the fact that a lack of evidence of susceptibility can be mistaken for a diagnosis of AR [25], in the case of TCBZ the comparative results would be presented as “inconclusive” in the treatment of the infection of the flock, following the four approaches (WAAVP method, Delta method, eggCounts method, and the customised Bayesian model). These criteria consider an efficacy target of 99% with a lower efficacy threshold of 95%. On the other hand, it should be noted that in this study we considered a value of 99% as the efficacy target, which could be applied to TCBZ, since its efficacy against mature forms can reach 100% [18]; however, this value does not seem to be so clear in initial studies on the efficacy of ABZ, with values around 95% [15]. However, some authors reported higher FECRT values for ABZ, such as 97% [62] or even 99% [52] against mature forms. Nevertheless, we are aware that further studies are needed to establish specific guidelines for detecting resistance to each of the different drugs in *Fasciola* spp.

## Figures and Tables

**Table 1:** Table of mean values in eggs per gram of faeces (epg) for albendazole (ABZ), oxfendazole (OXF), fenbendazole (FENB), and triclabendazole (TCBZ) and faecal egg count reduction percentages (% FECRT) for the customised Bayesian and eggCounts models. (D0–D14) = Day 0–day 14; (T–C) = treated control. N: number of animals.

Trial	N	Drug	Dose (mg/kg)	Mean Day 0 pt (epg)	Mean Day 14 pt (epg)	Mean Control (epg)	Mean Treated Day 14 pt (epg)	Customised Bayesian Model [42,43]		eggCounts Model [38,39]	
				Paired		Unpaired		%FECRT (D0–D14) Paired	%FECRT (T–C) Unpaired	%FECRT (D0–D14) Paired	%FECRT (T–C) Unpaired
**T1**	10	ABZ	**7.5**	78	29	107	29	56.0	63.0	64.0	75.3
	10	OXF	**5**	113	28	107	28	65.0	66.0	75.9	74.4
	10	OXF	**30**	74	21	107	21	57.0	66.0	73.1	82.6
**T2**	19	FENB	**10**	—	—	819	265	—	65.0	—	68.2
**T3**	28	TCBZ	**10**	44	1	—	—	94.0	—	97.6	—

**Table 2:** Faecal egg count reduction (FECR) estimates with 90% confidence intervals (CIs) for albendazole (ABZ), oxfendazole (OXF), fenbendazole (FENB), and triclabendazole (TCBZ). Using the different methodologies, the 90% CIs were calculated. For sheep, the grey zone is located between 95% and 99% FECR. (*) indicates “*resistant*” results, (♦) indicates “*inconclusive*”, and (●) indicates “*reduced efficacy*”. N: number of animals.

Trial	N	Drug	Dose (mg/kg)	Customised Bayesian Model [42,43]		eggCounts Model [38,39]		Delta Method [40]		WAAVP Method [26,41]	
				90% CI FECR (D0–D14) Paired (%)	90% CI FECR (T–C) Unpaired (%)	90% CI FECR (D0–D14) Paired (%)	90% CI FECR (T–C) Unpaired (%)	90% CI FECR (D0–D14) Paired (%)	90% CI FECR (T–C) Unpaired (%)	90% CI FECR (D0–D14) Paired (%)	90% CI FECR (T–C) Unpaired (%)
**T1**	10	ABZ	**7.5**	27–84 *	31–93 *	40.2–79.6 *	30.8–91.6 *	32.8–85.7 *	48.8–90.5 *	16.5–84 *	37.6–88.5 *
	10	OXF	**5**	39–89 ^●^	41–92 ^●^	58.7–86 ^●^	29.4–92.8 ^●^	50.7–91.8 ^●^	48.8–90.8 ^●^	34.7–90.4 ^●^	37–88.8 ^●^
	10	OXF	**30**	18–91 ^●^	30–98 ^●^	50.6–85.4 ^●^	39.5–96.3 ^●^	38.7–92.7 ^●^	56.7–95.4 ^●^	14.1–90.5 ^●^	41.8–93.3 ^●^
**T2**	19	FENB	**10**	—	54–77 ^●^	—	47.7–80.5 ^●^	—	52.9–79.9 ^●^	—	54.1–77.1 ^●^
**T3**	28	TCBZ	**10**	88–100 ^♦^	—	92.1–99.7 ^♦^	—	92.5–99.9 ^♦^	—	85.6–99.6 ^♦^	—

## Data Availability

The raw data supporting the conclusions of this article will be made available by the authors on request.
